# Does plate position influence the outcome in midshaft clavicular fractures? A multicenter analysis

**DOI:** 10.1007/s00068-023-02400-y

**Published:** 2024-01-17

**Authors:** Isabelle Ruth Buenter, Valerie Kremo, Frank Johannes Paulus Beeres, Nicole Maria van Veelen, Beat Galliker, Bjoern-Christian Link, Reto Babst, Hans-Christoph Pape, Bryan Joost Marinus van de Wall

**Affiliations:** 1 Department of Orthopaedic and Trauma Surgery, Cantonal Hospital Lucerne, Spitalstrasse, CH-6000 Lucerne, Switzerland; 2 Department of Surgery, Cantonal Hospital Lucerne in Sursee, Spitalstrasse 16A, CH-6210 Sursee, Switzerland; 3 Department of Orthopaedic and Trauma Surgery, Faculty of Health Science and Medicine University of Lucerne, Cantonal Hospital Lucerne, Spitalstrasse, CH-6000 Lucerne, Switzerland; 4 Department of Trauma, University Hospital Zurich, Raemistrasse 100, CH-8091 Zurich, Switzerland

**Keywords:** Midshaft clavicular fracture, Clavicle fracture, Plate position, Hardware irritation

## Abstract

**Purpose:**

To date, it remains unclear whether superior or anterior plating is the best option for treating midshaft clavicular fractures. The aim of this study was to compare both techniques with regard to the incidence of implant removal due to implant irritation, risk of complications, time to union, and function.

**Methods:**

In this retrospective cohort study, all midshaft clavicular fractures treated operatively between 2017 and 2020 in two hospitals in Switzerland were analyzed. The participating hospitals differed with regard to their standard practice; one offered superior plating only, while the other predominantly employed an anterior plate. The primary outcome was the incidence of implant removal for irritation. Secondary outcomes were time to union, complications, re-interventions, and range of motion during the follow-up period of at least 6 months.

**Results:**

In total, 168 patients were included in the study of which 81 (48%) received anterior plating and 87 (52%) superior plating. The overall mean age was 45 years (SD 16). There was no significant difference between anterior and superior plating with regard to implant removal (58.5% versus 57.1%, *p* = 0.887), infection (5.7% versus 1.8%, *p* = 0.071), and time to union (median 48 weeks versus 52 weeks, *p* = 0.643). Data on range of motion were available in 71 patients. There was no significant difference in anteflexion (median 180 degrees anterior versus 180 degrees superior) and abduction (median 180 degrees anterior versus 180 degrees superior) between the two groups.

**Conclusion:**

This retrospective cohort study did not find sufficient evidence to recommend one implant position over the other for midshaft clavicular fractures with regard to removal due to irritation. Time to union was similar and Infections were equally rare in both groups. Notably, a considerable number of patients in both groups had their implants removed due to irritation. Larger prospective studies are needed to determine how much plate position contributes to the occurrence of irritation and whether other patient or implant-related factors might play a role. Until this is clarified, implant position should be based on surgeons preference and experience.

**Supplementary Information:**

The online version contains supplementary material available at 10.1007/s00068-023-02400-y.

## Introduction

Clavicular fractures represent 2.6% of all fractures. With a prevalence of 44%, they represent the most common fractures of the shoulder girdle [1]. Approximately two thirds are located in the midshaft portion of the clavicle. The most appropriate treatment (operative or non-operative) for these fractures remains a point of debate. However, in the past years, surgical fixation has (re)gained popularity due to its superior results. The most recent meta-analysis showed a shorter time to bone union in the surgical group (mean difference -2.83 weeks, 96% CI − 4.59 to − 1.07; *p* = 0.002), a lower risk of non-union, mal-union and implant failure (risk ratio 0.21, 95% CI 0.1–0.42; *p* =  < 0.001), and improved Disabilities of the Arm, Shoulder and Hand (DASH) score (standard mean difference − 0.22, 95% CI − 0.36 to − 0.07, *p* = 0.003) [2].

Several options for the operative treatment of midshaft clavicular fractures exist, including plate fixation and intramedullary nailing. The use of intramedullary nailing (INM) is limited to simple two-part midshaft fractures. Its major disadvantage is implant migration and irritation (40% after INM versus 14% after plate fixation) leading to high removal rates (73% after INM versus 38% for plate fixation) [3, 4]. As fractures in the clavicle frequently are multifragmentary, plate fixation remains the most widely used technique [3].

When using a plate, the position is a matter of debate. It can either be positioned superiorly or anteriorly on the surface of the clavicle [5, 6]. The anterior plate position creates a biomechanically stronger construct allowing for longer screws and potentially prevents iatrogenic vascular injury [7]. Additionally, the anterior position has more soft tissue coverage theoretically leading to less plate prominence and irritation [8–10]. Superior plates are easier to position, and less muscle needs to be detached to position the plate [11]. A major disadvantage of superior plates is that they, due to their prominent position directly under the skin, lead to more irritation and, consequently, implant removal [8, 12]. Evaluating which plate position is better with regard to removal rates is important as it may significantly reduce the healthcare burden and costs related to this second operation.

Several studies have been published on this topic. However, those that have been published either suffered from a small sample size (such is the case for randomized clinical trials) or suffered from confounding (in case of observational studies), making it difficult to draw solid conclusions. The present study represents a natural experiment with one of the largest sample sizes published to date. Natural experiments are observational studies in which patients are exposed to either the experimental or the control condition, and treatment allocation is determined by factors outside the control of the investigators (i.e., geographical location). The process governing treatment allocation arguably resembles that of randomization. In this particular study, treatment allocation is determined by the location of the trauma, which is a random occurrence, and treatment type (superior or anterior plating) is determined by the local hospital near the trauma site where the patient presents him- or herself. A detailed description of this design in the orthopedic trauma field of research has recently been published [13].

In this natural experiment study, our research question is to compare the rates of implant removal between patients who underwent superior plate fixation to those who underwent anterior plating for midshaft clavicular fractures. We aim to assess whether there is a significant difference in the need for implant removal due to irritation or discomfort.

## Methods

### Study population and setting

This retrospective natural experiment study was performed in two hospitals in Switzerland that differed with respect to the preferred operative technique used for treating midshaft clavicular fractures. One hospital (hospital A) had both anterior and superior plating at the surgeons disposal. All treating surgeons within hospital A had a strong preference for one particular implant and routinely used the same implant in their treated patients. Notably, the majority of surgeons used anterior plating as their standard of care. The other hospital (hospital B) only had superior plating at their disposal. In other words, treatment allocation was largely based on the geographical location of the patient and, within hospital A, the surgeon performing the operation [13].

The hospital records of hospitals A and B were searched for all patients with a diagnosis code for clavicular fracture between January 2017 and January 2020. Patient records were screened for type of treatment received. The following inclusion criteria were employed: midshaft clavicular fracture treated operatively using either a superior or anterior plate and age older than 18 years. Midshaft fractures were defined according the AO definition for midshaft fractures [14]. Exclusion criteria were all other types of clavicular fractures (medial and lateral), other operative treatment than anterior or superior plating, re-interventions for non-union and polytrauma patients [15].

The study was approved by the ethics committee EKNZ Switzerland (proposal number 2020–00625). This article was written according to the Strengthening the Reporting of Observational Studies in Epidemiology (STROBE) guidelines.

### Operative technique

The surgeries were all performed by or under direct supervision of a consultant orthopedic trauma surgeon. The patients received general anesthesia and were placed in either beach chair or supine position. As preoperative antibiotic prophylaxis, the patients received 2 g cefazolin. As previously described, the choice of implant (anterior of superior) was determined by geographical location and, within hospital A, the treating surgeon. Hospital A used LCP Synthes plates (anterior and superior) and in hospital B Stryker LCP Variax superior clavicular plates were used. The reduction of the fracture and positioning of the plate was performed under visual and fluoroscopic control, and after fracture fixation was complete, at least two final intra-operative images perpendicular to each other were stored in the hospital Picture Archiving and Communication System (PACS).

### Postoperative treatment

All patients followed a similar postoperative protocol in both hospitals with the only difference being that hospital A routinely performed postoperative X-rays (AP and a perpendicular tangential view) within the first 72 h after surgery. For hospital B, the intra-operative X-rays were sufficient [16]. These differences were the result of natural variation in postoperative protocols. Logically, as the choice of postoperative X-ray is not related to the choice of implant and also not related to either of the outcomes, it, by definition, cannot cause confounding.

For comfort an arm sling was provided during the first two weeks and patients were advised to start range of motion exercises as soon as possible. The recommendation was active motion for hand an elbow and assisted for the shoulder joint. During the first six weeks, neither weight bearing nor abduction and anteflexion of the arm greater than 90° were allowed. Six weeks, three months, and one year after surgery, all patients were seen in the outpatient clinic with a routine X-ray. If patients reported implant-related complaints at the one-year outpatient visit, they were offered an implant removal provided the fracture was completely healed. The implant removal was usually performed 18 months after the primary surgery.

### Baseline characteristics

All data were collected by three independent authors who were not involved in the initial treatment. Patients characteristics such as gender, age, The American Society of Anesthesiologists (ASA) Score, anticoagulation/antiplatelet medication, high/low energy trauma, polytrauma (Injury Severity Score > 16), smoking at the time of presentation, time from first radiograph until surgery, operation duration, and time of the operation (during versus out of office hours) were extracted from the hospital records [17, 18]. Monday–Friday 07:00–18:00 h were defined as office hours with the exception of holidays.

The fractures were all classified according to the OTA/AO-Classification using the X-rays at presentation [14]. The Gustilo–Anderson classification was used for grading open fractures [19].

Data including incision and position of plate were extracted from the operative reports. All postoperative images, discharge letters, and letters from the outpatient visits were screened for potential deviation from normal postoperative protocol such as need for re-intervention, changes in movement, weight-bearing restrictions, postoperative infections, timing, and reason for implant removal.

### Outcome

The primary outcome was the number of implant removals due to implant irritation, weather sensitivity, or the patients’ wish for removal. Only patients who completed at least one-year follow-up were included for this analysis.

Secondary outcomes were time to union, occurrence of complications, re-interventions for reasons other than implant irritation, and range of motion during a follow-up period of at least 6 months. Deep infection was defined based on the guidelines on the Centers for Disease Control (CDC) [20]. Non-union was defined as the absence of fracture consolidation six months after surgery with radiologic bridging callus visible or fading of the fracture lines on less than three of the four cortices [21, 22].

### Statistical analysis

Statistical software package SPSS 25.0 was used to analyze the data. Descriptive statistics were provided of all baseline characteristics and study endpoints. Continuous variables were described as means (with standard deviation) or medians (with range) depending on distribution of the variables. For categorical variables the counts and percentage were calculated.

Differences in baseline characteristics were analyzed using the Students *T* test for normally distributed continuous variables, Mann–Whitney *U* test for non-normally distributed continuous variables and Chi-square for categorical variables. Significant differences in baseline characteristics were considered to be potential confounders (data-driven selection of confounding variables). To account for confounding, a stratified analysis was performed of all outcome variables for each potential confounder. Stratification was chosen as the preferred method to deal with differences as it does not lead to loss of power (otherwise the case with matching) and it creates the possibility to calculate absolute percentages instead of only odds ratios or relative risks. The level of significance was set at a threshold of 0.05.

## Results

### Study population

**Fig. 1:**
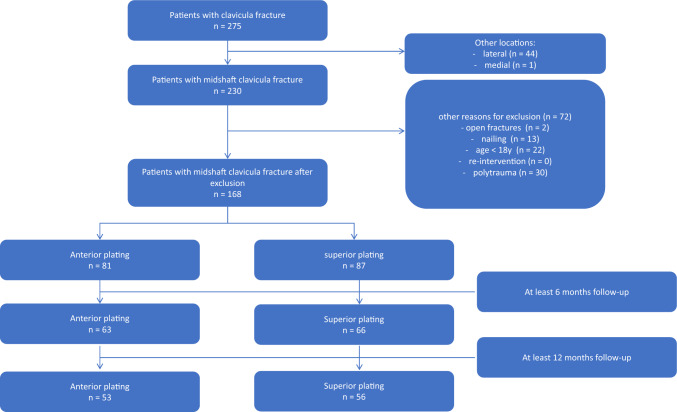
Flowchart of study population and number of patients available for primary and secondary analyses

Between January 2017 and January 2020, a total of 275 patients underwent plate fixation for clavicular fractures in the two hospitals. After applying the exclusion criteria, 168 patients could be included in the final analysis: 81 patients with anterior plating and 87 with superior plates (Fig. 1). Six- and twelve-month follow-up data were available in 129 and 109 patients, respectively. The other patients were lost to follow-up as they were either tourists who returned to their home country or did not present at the outpatient visit for unknown reasons.

### Baseline characteristics

Baseline characteristics for both treatment groups are described in Table 1. The only significant difference between the two treatment groups was the proportion of smokers (27.2% in the anterior versus 10.3% in the superior group) and diabetes mellitus (75% in the anterior versus 25% in the superior group).

**Table 1:** Baseline characteristics

Patient-related variable	Clavicular fractures (*n* = 168)	Anterior plate (*n* = 81)	Superior plate (*n* = 87)	*p* value
Sex distribution				0.320
Male	132 (78.6%)	61 (75.3%)	71 (81.6%)	
Female	36 (21.4%)	20 (24.7%)	16 (18.4%)	
Mean age (years)	45.18 Mean (18–85) SD 15.753 Median 45.5	45.36 Mean (18–85) SD 15.724 Median 45	45.02 Mean (18–81) SD 15.869 Median 46	
ASA				0.312
I	89 (53%)	38 (46.9%)	51 (58.6%)	
II	72 (42.9%)	39 (48.1%)	33 (37.9%)	
III	7 (4.2%)	4 (4.9%)	3 (3.4%)	
Diabetes	4 (2.4%)	3 (3.7%)	1 (1.1%)	0.278
Anticoagulant/antiplatelet medication				0.515
None	157 (93.5%)	75 (92.6%)	82 (94.3%)	
Phenprocoumon	1 (0.6%)	1 (1.2%)	0	
Aspirin	8 (4.8%)	4 (4.9%)	4 (4.6%)	
New oral anticoagulants	2 (1.2%)	1 (1.2%)	1 (1.1%)	
Smoking				0.005
Yes	31 (18.5%)	22 (27.2%)	9 (10.3%)	
No	137 (81.5%)	59 (72.8%)	78 (89.7%)	
AO-classification				0.723
A	45 (26.8%)	24 (29.6%)	21 (24.1%)	
B	45 (26.8%)	21 (25.9%)	24 (27.6%)	
C	78 (46.4%)	36 (44.4%)	42 (48.3%)	

Table 2 demonstrates the baseline characteristics for both treatment groups stratified per hospital. Notable is that the baseline characteristics are comparable between hospitals A and B and within hospital A for the anterior and superior plating group.

**Table 2:** Baseline characteristics—hospitals A and B

Patient-related variable	Hospital A Anterior plate (*n* = 81)	Hospital A Superior plate (*n* = 33)	Hospital B Superior plate (*n* = 54)	*p* value
Sex distribution				0.565
Male	61 (75.3%)	30 (90.9%)	41 (75.9%)	
Female	20 (24.7%)	3 (9.1%)	13 (24.1%)	
Mean age (years)	45.36 Mean (18–85) SD 15.724 Median 45	45.64 Mean (18–79) SD 16.282 Median 45	44.65 Mean (18–81) SD 15.754 Median 47	0.762
ASA				0.057
I	38 (46.9%)	16 (48.5%)	35 (64.8%)	
II	39 (48.1%)	17 (51.5%)	16 (29.6%)	
III	4 (4.9%)	0	3 (5.6%)	
IV	0	0	0	
Diabetes	3 (3.7%)	0	1 (1.9%)	0.757
Anticoagulant/antiplatelet medication				
None	75 (92.6%)	29 (87.9%)	53 (98.1%)	0.684
Phenprocoumon	1 (1.2%)	0	0	
Aspirin	4 (4.9%)	3 (9.1%)	1 (1.9%)	
New oral anticoagulants	1 (1.2%)	1 (3%)	0	
Smoking				0.003
Yes	22 (27.2%)	6 (18.2%)	3 (5.6%)	
No	59 (72.8%)	27 (81.8%)	51 (94.4%)	
AO-classification				0.861
A	24 (29.6%)	8 (24.2%)	13 (24.1%)	
B	21 (25.9%)	9 (27.3%)	15 (27.8%)	
C	36 (44.4%)	16 (48.5%)	26 (48.1%)	

### Operative data

Operative data are described in Table 3. Surgery was more frequently performed in out of office hours among the group of patients who received a superior plate. Other characteristics were equally distributed among the two treatment groups.

**Table 3:** Operative data

Perioperative findings	Clavicular fractures (*n* = 168)	Anterior plate (*n* = 81)	Superior plate (*n* = 87)	*p* value
Mean time first X-ray till surgery (days)	Median 2 (0–29)	Median 4 (0–24)	Median 1 (0–29)	
Operations out of office	43 (25.6%)	15 (18.5%)	28 (32.2%)	0.043
Cut-suture time (minutes)	Median 111.5 (50–354)	Median 116 (67–354)	Median 108 (50–231)	
Skin incision/approach				0.211
Skinline	139 (82.7%)	64 (79%)	75 (86.2%)	
Subclavicular	9 (5.4%)	4 (4.9%)	5 (5.7%)	
MIPO	4 (2.4%)	4 (4.9%)	0	
Coup de sabre	15 (8.9%)	8 (9.9%)	7 (8%)	
Unknown	1 (0.6%)	1 (1.2%)	0	

### Primary outcome

One-hundred and nine patients had a one-year follow-up. There was no significant difference in number of implant removals between the anterior (58.5%) and superior (57.1%) plating group (p-value 0.887).

### Secondary outcome

All secondary outcomes are described in Tables 4 and 5. Four patients had a complication that required operative treatment. Three (5.7%) in the anterior and one (1.8%) in the superior group (*p* value 0.07). All four patients had a deep surgical site infection (deep SSI) [20].

**Table 4:** Outcomes on one-year follow-up—primary outcome

Outcome—postoperative findings	Clavicular fractures (*n* = 109)	Anterior plate (*n* = 53)	Superior plate (*n* = 56)	*p* value
Re-intervention after primary operation				0.183
Implant removal (Implant irritation)	66 (57.8%)	31 (58.5%)	32 (57.1%)	0.887
Deep SSI	4 (3.7%)	3 (5.7%)	1 (1.8%)	0.071

**Table 5:** Outcomes on six-month follow-up—secondary outcome

Outcome—postoperative findings	Clavicular fractures (*n* = 129)	Anterior plate (*n* = 63)	Superior plate (*n* = 66)	*p* value
Range of motion between 6 and 12 months				
Anteflexion	180 Median (120–180) IQR 0	180 Median (120–180) IQR 8	180 Median (150–180) IQR 0	0.49
Abduction	180 Median (90–180) IQR 0	180 Median (90–180) IQR 5	180 Median (150–180) IQR 0	0.195
Time to union (weeks)				0.643
	51 Median (8–110) IQR 32	48 Median (10–79) IQR 30	52 Median (8–110) IQR 28	
Time to union (weeks) At least 6-month follow-up				0.526
	52 Median (10–110) IQR 25	49 Median (10–79) IQR 28	53 Median (13–110) IQR 18	
Non-union				0.587
Atrophic non-union	3 (3.2%)	1 (1.6%)	2 (3%)	

The first patient within the anterior plating group developed clinical signs of an infection three months after osteosynthesis. The samples that were taken during the operative revision confirmed a deep infection. In a second revision, the implant was removed and a re-osteosynthesis was performed.

The second patient within this group had an implant failure four weeks after primary surgery. He underwent re-osteosynthesis and cultures which were obtained during the revision were positive for bacteria. The third patient in the anterior plating group had an atrophic septic non-union diagnosed 40 weeks after primary surgery with clinical instability. He underwent re-osteosynthesis as well.

The one patient with superior plating had an implant failure 16 weeks after primary surgery also caused by a septic non-union. He also underwent re-osteosynthesis. All four patients were treated with antibiotics for three months.

There were three (3.2%) patients with aseptic complications (*p* value 0.587). All of them had a non-union which was treated conservatively due to a lack of symptoms. No other complications were seen in the study population.

In 71 patients the range of motion at least six months after surgery was described. The median anteflexion was 180 (120–180) degrees in the anterior and 180 (150–180) degrees in the superior group (*p* value 0.49). Median abduction was 180 (90–180) degrees in the anterior and 180 (150–180) degrees in the superior group (*p* value 0.195). There was no significant difference between the groups.

The median time until union was 49 (10–79) weeks in the anterior und 53 (13–110) weeks in the superior group (*p* value 0.526).

### Subgroup analysis

Stratified analyses for all outcomes were performed to account for differences in baseline and operative characteristics including smoking status, diabetes mellitus, and surgery outside office hours. These analyses are described in Supplementary Tables 7–12. No confounding effect of these variables was detected in the analyses between anterior and superior plating for both primary and secondary outcomes.

## Discussion

This retrospective natural experiment study comparing anterior to superior plating for midshaft clavicular fractures was not able to detect any difference in re-intervention rate, complications, time to union or range of motion. Notably, more than half of the patients with clavicular plates end up having their implant removed, mostly because of irritation. Fracture union was achieved in almost all patients and in the rare cases it did not heal, infection was the predominant cause.

The baseline characteristics were comparable in both groups. This supports our claim that choice of implant is a product of either geographical location and surgeons preference. All in all, this supports the natural experiment study design [13].

### Comparison with previous literature

This study represents one of the largest cohorts of patients with midshaft clavicular fractures to be evaluated with regard to plate position to date. Its novelty lies in the natural experiment design creating the opportunity to study a well-investigated problem in a new manner than previously employed (randomized clinical trials and retrospective cohort studies) [13].

Our findings are not fully in line with the most recent meta-analysis of Ai et al., which included four RCTs and eight observational studies on this topic [23]. This meta-analysis concluded that anterior plating had a shorter operation duration, lower intra-operative blood loss and faster time to union compared to superior plating. It must, however, be acknowledged that the absolute differences in blood loss (average 80 ml versus 100 ml) and operation time (69 versus 84 min) are small, and statistical significance is based on pooled analysis of the standardized mean difference, instead of the actual mean difference. Additionally, no pooled analysis was performed on time to union due to poor reporting in included studies. Nevertheless, the authors concluded that time to union was shorter for anterior plating based on the fact that two out of the seven studies reported such a difference.

All in all, on a statistical level, there are differences between the present study and the meta-analysis. However, from a clinical point of view, both studies may agree that differences, when detected, are rather small and insufficient to conclude superiority of one over the other.

The meta-analysis did not report on our primary outcome of interest, the rate of implant removal. A search in literature identified seven studies (one RCT, one prospective cohort study, and five retrospective observational studies) that directly compared both plating positions with each other and reported on implant removal due to irritation (Table 6) [8, 10, 24–28]. There is a significant amount of heterogeneity in removal rates between studies and between treatment groups. Reasons for this heterogeneity remain unclear but the fact that studies from Europe (including ours) have a higher removal rate in general (ranging from 37 to 61% for superior and 36 to 67% for anterior plating) than studies from the USA (ranging from 19 to 22.3% for superior and 5.9 to 9% for anterior plating) suggests that these differences might be caused by cultural factors and local education/conviction. In other words, it is not unlikely that the choice to remove a plate from the clavicle is not so much driven by its position but rather other factors (cultural differences, patient age, level of activity). Be it as it may, our removal rates are equally high as the study by Nolte which has a similar cultural background and demographic as the present study [28]. They found a removal rate of 67% for superior versus 61% for anterior plating which is comparable to the present study with a rate of 57.1% and 58.5%, respectively. They also did not detect any significant difference supporting our conclusion.

**Table 6:** Study comparison

Author	Year		Design	*N* patients	Removal rate (%)	
					Anterior	Superior
Formani [8]	2013	USA	Retro	105	9	19
Sohn [24]	2015	Korea	RCT	37	17	21
Hulsman [25]	2016	Netherlands	Retro	99	36	37
Serrano [26]	2017	USA	Retro	251	5.9	22.3
Sinkler [27]	2022	USA	Retro	175	8	19
Nolte [28]	2021	Germany	Retro	79	67	61
Mullis [10]	2023	USA	Prospective	412	6.2	7.1

## Limitations

Although this study has a natural experiment design and control for measured confounders was possible, unmeasured confounding still remains an issue. The quasi-randomization mechanism in natural experiments minimizes the risk, but will not be as effective as true randomization in trials that basically nullifies the risk if done properly. Additionally, due to the retrospective nature, information bias might have occurred. A good example is the smoking status. The unequal distribution between the two treatment groups was probably caused by inadequate documentation. Notably, stratified analysis showed that it did not affect the results of the study. This also applies for other differences in baseline characteristics such as diabetes mellitus and surgery performed outside of office hours.

We had a considerable number of patients who did not show up for the 12-month outpatient visit. As reasons also remain unknown, we cannot say whether this was random or non-random loss-to-follow-up. Therefore, selection bias cannot be excluded.

Lastly, this study focused on the plate position on the clavicle. There are multiple plates from different manufacturers (e.g., Synthes, Stryker, Arthrex) currently on the market. Each plate has their own dimensions. Although the differences in dimensions are small, we suspect the plate thickness (supplementary Table 13) may have more influence on the occurrence of irritation than plate position. However, this was not evaluated in the current study.

## Conclusion

This retrospective cohort study did not find sufficient evidence to recommend one implant position over the other for midshaft clavicular fractures with regard to removal due to irritation. Time to union was similar and Infections were equally rare in both groups. Notably, a considerable number of patients in both groups had their implants removed due to irritation. Larger prospective studies are needed to determine how much plate position contributes to the occurrence of irritation and whether other patient or implant-related factors might play a role. Until this is clarified, implant position should be based on surgeons preference and experience.

### Supplementary Information

Below is the link to the electronic supplementary material.

Supplementary file1 (DOCX 22 KB)

## Data Availability

Is available and will be provided if needed.
