# Supplementary material for: Does plate position influence the outcome in midshaft clavicular fractures? A multicenter analysis
Source: Eur J Trauma Emerg Surg. 2024 Jan 17;50(3):1023–31. doi: 10.1007/s00068-023-02400-y (PMC11249707; doi:10.1007/s00068-023-02400-y)
Supplement: Supplementary file 1 — Supplementary file1 (DOCX 22 KB) [file 68_2023_2400_MOESM1_ESM.docx]

# Supplementary Tables

*Table 7: Outcomes smokers*

| Outcome – postoperative Findings | | Clavicular fractures (n = 31) | Anterior plate (n = 22) | Superior plate (n = 9) | p-value |
| --- | --- | --- | --- | --- | --- |
| Re-intervention after primary operation | | Total 21 | Total 14 | Total 7 | 0.190 |
|  | Implant removal (Implant irritation)  **After 1 year** | 14 (66.7%) | 8 (57.1%) | 6 (85.7%) | 0.190 |
|  | Deep SSI  **After 1 year** | 0 | 0 | 0 |  |
| Range of Motion between 6-12 months | | n = 15 | n = 8 | n = 7 |  |
|  | Anteflexion | 180 Median (135-180)  IQR 10 | 180 Median (135-180)  IQR 8 | 175 Median (150-180)  IQR 25 |  |
|  | Abduction | 180 Median (110-180)  IQR 8 | 180 Median (110-180)  IQR 8 | 177.5 Median (170-180)  IQR 8 |  |
| Time to union (weeks) | | n = 25 | n = 17 | n = 8 | 0.311 |
|  |  | 39 Median (11-104)  IQR 27 | 39 Median (11-79)  IQR 43 | 36 Median (19-104)  IQR 14 |  |
| Time to union (weeks)  At least 6 Months Follow up | | n = 23 | n = 16 | n = 7 | 0.308 |
|  |  | 39 Median (11-104)  IQR 27 | 44 Median (11-79)  IQR 45 | 39 Median (25-104)  IQR 12 |  |
| Non union | | n = 23 | n = 16 | n = 7 |  |
|  | Atrophic non-union | 0 | 0 | 0 |  |

*Table 8: Outcomes non-smokers*

| Outcome – postoperative Findings | | Clavicular fractures (n = 137) | Anterior plate (n = 59) | Superior plate (n = 78) | p-value |
| --- | --- | --- | --- | --- | --- |
| Re-intervention after primary operation | | Total 88 | Total 39 | Total 49 | 0.031 |
|  | Implant removal (Implant irritation)  **After 1 year** | 49 (55.7 %) | 23 (59%) | 26 (53.1%) | 0.579 |
|  | Deep SSI  **After 1 year** | 4 (4.5%) | 3 (7.7%) | 1 (2.0%) |  |
| Range of Motion between 6-12 months | | n = 59 | n = 25 | n = 34 |  |
|  | Anteflexion | 180 Median (120-180)  IQR 0 | 180 Median (120-180)  IQR 8 | 180 Median (150-180)  IQR 0 |  |
|  | Abduction | 180 Median (90-180)  IQR 0 | 180 Median (90-180)  IQR 5 | 180 Median (150-180)  IQR 0 |  |
| Time to union (weeks) | | n = 110 | n = 50 | n = 60 | 0.623 |
|  |  | 51 Median (8-110)  IQR 13 | 48 Median (10-79)  IQR 24 | 53 Median (8-110)  IQR 12 |  |
| Time to union (weeks)  At least 6 Months Follow up | | n = 99 | n = 46 | n = 53 | 0.511 |
|  | | 52 Median (10-110)  IQR 11 | 49.5 Median (10-79)  IQR 20 | 54 Median (13-110)  IQR 13 |  |
| Non union | | n = 106 | n = 47 | n = 59 |  |
|  | Atrophic non-union | 3 (2.8%) | 1 (2.1%) | 2 (3.4%) |  |

Table 9: Outcomes Diabetes

| Outcome – postoperative Findings | | Clavicular fractures (n = 4 ) | Anterior plate (n = 3 ) | Superior plate (n = 1) | p-value |
| --- | --- | --- | --- | --- | --- |
| Re-intervention after primary operation | | Total = 3 | Total =1 | Total = 0 |  |
|  | Implant removal (Implant irritation)  **After 1 year** | 1 (33.3%) | 1 | 0 |  |
|  | Deep SSI  **After 1 year** | 0 | 0 | 0 |  |
| Range of Motion between 6-12 months | | n = 0 | n = 0 | n = 0 |  |
|  | Anteflexion |  |  |  |  |
|  | Abduction |  |  |  |  |
| Time to union (weeks) | | n = 3 | n = 3 |  |  |
|  |  | 54 Median (48-70) | 54 Median (48-70) |  |  |
| Time to union (weeks)  At least 6 Months Follow up | | n = 3 | n = 3 |  |  |
|  |  | 54 Median (48-79) | 54 Median (48-79) |  |  |
| Non union | | 0 | 0 | 0 |  |
|  | Atrophic non-union | 0 | 0 | 0 |  |

Table 10: Outcomes non-Diabetes

| Outcome – postoperative Findings | | Clavicular fractures (n = 164) | Anterior plate (n = 78) | Superior plate (n = 86) | p-value |
| --- | --- | --- | --- | --- | --- |
| Re-intervention after primary operation | | Total = 102 | Total = 50 | Total = 56 | 0.173 |
|  | Implant removal (Implant irritation)  **After 1 year** | 69 (57.8%) | 30 (60%) | 32 (57.1%) | 0.766 |
|  | Deep SSI  **After 1 year** | 2 (1.9%) | 2 (4%) | 0 | 0.063 |
| Range of Motion between 6-12 months | | n = 71 | n = 33 | n = 38 |  |
|  | Anteflexion | 180 Median (120-180)  IQR 0 | 180 Median (120-180)  IQR 8 | 180 Median (150-180)  IQR 0 | 0.490 |
|  | Abduction | 180 Median (90-180)  IQR 0 | 180 Median (90-180)  IQR 5 | 180 Median (150-180)  IQR 0 | 0.195 |
| Time to union (weeks) | | n = 135 | n = 64 | n = 68 | 0.643 |
|  |  | 51 Median (8-110)  IQR 24 | 48 Median (10-79)  IQR 28 | 52 Median (8-110)  IQR 22 |  |
| Time to union (weeks)  At least 6 Months Follow up | | n = 119 | n = 59 | n = 60 | 0.526 |
|  |  | 52 Median (10-110)  IQR 18 | 47.5 Median (10-79)  IQR 25 | 53 Median (13-110)  IQR 13 |  |
| Non union | | n = 123 | n = 60 | n = 64 | 0.587 |
|  | Atrophic non-union | 3 (2.4%) | 1 (1.7%) | 2 (3%) |  |

Table 11: Outcomes OP out of office hours

| Outcome – postoperative Findings | | Clavicular fractures (n = 43) | Anterior plate (n = 15) | Superior plate (n = 28) | p-value |
| --- | --- | --- | --- | --- | --- |
| Re-intervention after primary operation | | Total = 29 | Total = 10 | Total = 19 | 0.523 |
|  | Implant removal (Implant irritation)  **After 1 year** | 16 (55.2%) | 5 (50%) | 11 (57.9%) | 0.684 |
|  | Deep SSI  **After 1 year** | 1 (3.4 %) | 1 (10%) | 0 | 0.161 |
| Range of Motion between 6-12 months | | n = 15 | n = 6 | n = 9 |  |
|  | Anteflexion | 180 Median (120-180)  IQR 0 | 180 Median (120-180)  IQR 49 | 180 Median (180-180)  IQR 0 | 0.177 |
|  | Abduction | 180 Median (110-180)  IQR 0 | 180 Median (110-180)  IQR 70 | 180 Median (180-180)  IQR 0 | 0.063 |
| Time to union (weeks) | | n = 33 | n = 13 | n = 20 |  |
|  |  | 52 Median (12-110)  IQR 9 | 49 Median (12-77)  IQR 27 | 52.5 Median (13-110)  IQR 11 | 0.270 |
| Time to union (weeks)  At least 6 Months Follow up | | n = 30 | n = 11 | n = 19 |  |
|  |  | 52 Median (12-110)  IQR 14 | 50 Median (12-77)  IQR 27 | 52 Median (13-110)  IQR 11 | 0.264 |
| Non union | | n = 31 | n = 11 | n=20 |  |
|  | Atrophic non-union | 0 | 0 | 0 |  |

Table 12: Outcomes OP during office hours

| Outcome – postoperative Findings | | Clavicular fractures (n = 125) | Anterior plate (n = 66) | Superior plate (n = 59) | p-value |
| --- | --- | --- | --- | --- | --- |
| Re-intervention after primary operation | | Total = 80 | Total = 43 | Total = 37 |  |
|  | Implant removal (Implant irritation)  **After 1 year** | 47 (58.8%) | 26 (60.5%) | 22 (56.8%) | 0.963 |
|  | Deep SSI  **After 1 year** | 3 (3.8%) | 2 (4.7%) | 1 (2.7%) | 0.731 |
| Range of Motion between 6-12 months | | n = 55 | n = 27 | n = 29 |  |
|  | Anteflexion | 180 Median (140-180)  IQR 0 | 180 Median (140-180) IQR 5 | 180 Median (150-180)  IQR 0 | 0.604 |
|  | Abduction | 180 Median (90-180)  IQR 0 | 180 Median (90-180)  IQR 0 | 180 Median (150-180)  IQR 0 | 0.476 |
| Time to union (weeks) | | n = 102 | n = 54 | n = 48 |  |
|  |  | 50 Median (8-105)  IQR 31 | 48 Median (10-79)  IQR 28 | 51.5 Median (8-105)  IQR 28 | 0.752 |
| Time to union (weeks)  At least 6 Months Follow up | | n = 92 | n = 51 | n = 41 |  |
|  |  | 51 Median (10-105)  IQR 25 | 48 Median (10-79)  IQR 27 | 54 Median (13-105)  IQR 14 | 0.6161 |
| Non union | | n = 98 | n = 51 | n = 46 |  |
|  | Atrophic non-union | 3 (3.1%) | 1 (1.9%) | 2 (4.3%) | 0.491 |

Table 13: Plate Thickness

| Plate |  |
| --- | --- |
| Stryker Variax 2.0 superior | 3.3 mm |
| Synthes 3.5mm LCP superior | 3 mm |
| Synthes 2.7/3.5mm VA-LCP anterior | 2.5 mm |
